# Composition of Royal Jelly (RJ) and Its Anti-Androgenic Effect on Reproductive Parameters in a Polycystic Ovarian Syndrome (PCOS) Animal Model

**DOI:** 10.3390/antiox9060499

**Published:** 2020-06-07

**Authors:** Norhamidar Ab Hamid, Ainul Bahiyah Abu Bakar, Anani Aila Mat Zain, Nik Hazlina Nik Hussain, Zaidatul Akmal Othman, Zaida Zakaria, Mahaneem Mohamed

**Affiliations:** 1 Department of Physiology, School of Medical Sciences, Universiti Sains Malaysia, Kubang Kerian 16150, Malaysia; nah15_med043@student.usm.my (N.A.H.); ainul@usm.my (A.B.A.B.); zaidaakmal@unisza.edu.my (Z.A.O.); zaida_zakaria@student.usm.my (Z.Z.); 2 Department of Pathology, School of Medical Sciences, Universiti Sains Malaysia, Kubang Kerian 16150, Malaysia; ailakb@usm.my; 3 Unit of Women’s Health Development, School of Medical Sciences, Universiti Sains Malaysia, Kubang Kerian 16150, Malaysia; hazlinakck@usm.my; 4 Unit of Physiology, Faculty of Medicine, Universiti Sultan Zainal Abidin, Kuala Terengganu 20400, Malaysia; 5 Unit of Integrative Medicine, School of Medical Sciences, Universiti Sains Malaysia, Kubang Kerian 16150, Malaysia

**Keywords:** polycystic ovary syndrome, royal jelly, immature rats, antioxidant, LC–MS

## Abstract

Royal jelly (RJ) has been shown to contribute its positive effects upon imbalance in the reproductive system. However, it remains unknown as to whether RJ has an anti-androgenic effect on reproductive parameters in a polycystic ovarian syndrome (PCOS) animal model. Composition of RJ was assessed by phytochemical screening and the LC–MS method. Forty immature female rats (3 weeks, 40–50 g) were randomly divided into five groups (*n* = 8 per group), i.e., control, testosterone (T), T+100RJ (100 mg/kg/day), T+200RJ (200 mg/kg/day RJ), and T+400RJ (400 mg/kg/day RJ) groups. Hyperandrogenism was induced by daily subcutaneous injection of T propionate for 3 weeks, followed by oral RJ for 4 weeks. The T+200RJ group had a significantly higher follicle-stimulating hormone level, and significantly lower luteinizing hormone, testosterone, and estradiol levels in comparison to the T group. Malondialdehyde level and glutathione peroxidase activity were significantly lower, while total antioxidant capacity level was significantly higher in the T+200RJ group compared to the T group. Histologically, the T+200RJ group showed recovery of various stages of ovarian follicular development. RJ at 200 mg/kg/day for 4 weeks significantly improved reproductive parameters in PCOS rats partly due to its anti-androgenic effect through antioxidant action and probably due to modulation on estrogenic activity, which needs further study to evaluate its exact mechanism of action.

## 1. Introduction

Polycystic ovarian syndrome (PCOS) is the most common female endocrine disorder that affects women at active reproductive age. The prevalence of PCOS was estimated at about 12.6% [1] in Malaysia and at 5–16% for women of different ethnicities worldwide [2]. The Rotterdam Consensus Criteria for diagnosis of PCOS depends upon the presence of at least two of the following symptoms: oligo-/or anovulation (ovulatory dysfunction), features of hyperandrogenism, and polycystic ovaries [3]. Hyperandrogenism, or surplus of circulating androgens, is one of the most important characteristics of polycystic ovary syndrome (PCOS) which results in disturbance of ovarian function and female fertility. The excess androgen levels can modify gonadotropin-induced progesterone and estrogen synthesis in the follicles [4], consequently leading to physical and physiological disturbance such as hirsutism, menstrual abnormality, infertility, and glucose intolerance. Studies also have reported that oxidative stress (OS) is closely linked to pathological features of PCOS, and individuals with PCOS have imbalance in antioxidant status [5]. Conventional hormonal replacement therapy for PCOS patients seems to produce undesirable side effects such as breast tenderness, vaginal spotting, and hypertension [6]. Therefore, it is crucial to provide and explore new alternative therapies to retard hyperandrogenism with minimal side effects.

RJ (royal jelly) or bee’s milk is produced by the hypopharyngeal glands of the worker honeybees and is composed of a yellowish-white acidic secretion with a slightly pungent odor and taste [7]. It is considered a vital product among honeybee production with high biological and nutritional properties. Generally, it consists of water (60–70%), proteins (9–18%), sugars (7–18%), lipids (3–8%), minerals, vitamins, and essential amino acids [8]. It possesses some biological properties such as antibacterial [9], anti-cancer [10], immunomodulatory [11], and antioxidant [9,12,13] properties. It also improves menopausal symptoms [14], increases fertility [15], improves reproductive performance, and reduces OS status in male rabbits [16]. Studies have shown that intake of RJ increases progesterone hormone [17] and pregnancy rate [18] in female sheep. RJ has also been shown to modulate estrogenic activity by interaction with estrogen receptors (ERs) and to enhance mRNA expression of estrogen-responsive genes [19]. 10-Hydroxy-trans-2-decenoic acid (10-HDA), 10-hydroxydecanoic acid (HDAA), trans-2-decenoic acid, and 24-methylenecholesterol [20] are compounds that are present in RJ and have been identified as the most effective ligand-binding assays for ERs. To date, no study has been performed on the possible anti-androgenic effects of RJ on reproductive parameters in a PCOS animal model. Therefore, in the current study, we evaluated various doses of RJ for any effects on hormonal profile, estrus cycle, oxidant-antioxidant status, and ovarian histology, in relation to the presence of its phytochemical and bioactive compounds.

## 2. Materials and Methods

### 2.1. Royal Jelly

RJ from honeybee (*Apis mellifera*) was purchased from a local beekeeper in Johor, Malaysia. It was collected during the period of February to June (dry season) and stored at −80 °C. On the basis of the body surface area normalization method, the dose of RJ (100 mg/kg in rat) for the animal study was relative to the amount of fresh RJ traditionally consumed in humans, which is 1 g/kg body weight [21]. Therefore, the doses used in the present study were 100 mg/kg (as a low dose), 200 mg/kg (as a medium dose), and 400 mg/kg (as a high dose). RJ was freshly prepared by suspending it in distilled water to make 0.5 mL suspension, and it was then administered to the rats by oral gavage.

### 2.2. Phytochemical Screening and Liquid Chromatography–Mass Spectrometry (LC–MS) Analysis

Phytochemical screening test was performed using previous standardized methods to evaluate the presence for alkaloids, phenols [22], flavonoids [23], glycosides, resins, saponins, tannins, terpenoids, and xanthoproteins [24]. Liquid chromatography–mass-spectrometry (LC–MS) analysis was performed to analyze the presence of non-volatile phytochemical compounds. It was performed using Finnegan Surveyor plus HPLC instrument (Shimadzu-Hitachi, Kyoto, Japan) equipped with a diode array detection (DAD) (Shimadzu, Tokyo, Japan) and coupled to a MS (Shimadzu, Tokyo, Japan). The chromatographic system consisted of quaternary pump, auto-sampler, degasser, photodiode-array detector, and automatic thermostatic column compartment. A total of 10 mg of RJ was filtered through a 0.2 µm nylon membrane at a rate of 1 mL/min, then 200 µL/min was split out to the mass spectrometer. The experiment was performed using helium as collision gas at an energy rate of 25–40 eV. The peak mass spectra in mass per charge ions (*m/z*) were acquired for the possible compound names in the range of 200–600 nm and identification of compounds was performed by using ReSPect (RIKEN MSn spectral database for phytochemicals, Yokohama, Japan).

### 2.3. Animal Experimental Design

Forty immature female Sprague Dawley rats (40–50 g; 3 weeks old) were acquired from the Animal Research and Service Centre (ARASC), Universiti Sains Malaysia. Adaptation was allowed for all rats for at least 4 days in laboratory prior to the experiment. The rats were kept under ambient temperature (22 ± 2 °C) with 12:12 h light/dark cycle, water was provided ad libitum, and they were fed a commercial rat diet (Gold Coin Sdn. Bhd., Selangor, Malaysia). The rats were divided by a simple randomization into control (normal healthy rats; *n* = 8) and testosterone (T)-treated (*n* = 32) groups. Hyperandrogenism was induced in the T-treated group by daily subcutaneous (SC) injection of T propionate in olive oil (10 mg/kg) for 3 weeks while the control group received an equivalent amount of SC olive oil [25]. After 3 weeks, the animals in each group were daily treated for 4 weeks as follows:

(a) Control group: given SC olive oil (10 mg/kg bodyweight) and oral distilled water (0.5 mL);
(b) T group: given SC testosterone and oral distilled water;
(c) T+100RJ group: given SC testosterone and oral 100 mg/kg RJ;
(d) T+200RJ group: given SC testosterone and oral 200 mg/kg RJ;
(e) T+400RJ group: given SC testosterone and oral 400 mg/kg RJ.

At the end of experimental period, the animals were fasted overnight and anesthetized with intraperitoneal 90 mg/kg ketamine and 5 mg/kg xylazine during diestrus phase. Rats were sacrificed and blood was collected via posterior vena cava for determination of reproductive hormones. The ovary was dissected out for determination of oxidant/antioxidant status and histological assessment. The animal experiment was conducted in accordance with the Guide for the Care and Use of Laboratory Animals of Universiti Sains Malaysia, and this study was approved by the Animal Ethic Committee, Universiti Sains Malaysia (USM/Animal Ethics Approval, 2013 (90) (505)).

### 2.4. Measurement of Reproductive Hormones

Blood sample was centrifuged, and the separated serum was used to determine reproductive hormones (testosterone (T), estradiol (E_2_), luteinizing hormone (LH), and follicle-stimulating-hormone (FSH)) levels using commercially available kits (Cloud clone Corp., Houstan, TX, USA, and Qayee-Bio Life Science Co. LTD., Shanghai, China).

### 2.5. Evaluation of Estrus Cycle

During the treatment, estrus cycle was assessed daily by vaginal smear for 4 weeks every morning between 9.00 a.m. and 10.00 a.m. A small amount of 0.9% normal saline was introduced into the entrance of vaginal canal and gently flushed. The suspension was placed on a glass slide using a blunt-ended glass dropper tip to assess the presence of leucocytes, cornified epithelial cells, and nucleated epithelial cells. The slide was closed with cover slip and examined at 10× and 40× magnifications under a light microscope (Leica DM750, LEICA, Wetzlar, Germany). The evaluation of estrus cycle was determined on the basis of the proportion of the cells [26].

### 2.6. Measurement of Oxidant-Antioxidant Status

The right ovary was removed, washed in ice-cold normal saline, and immediately homogenized with phosphate-buffered saline. After being centrifuged (Avanti J-HC, Beckman Coulter, Brea, CA, USA) at 4000 rpm for 15 min, the supernatant was collected and stored at −80 °C until further use for analysis of oxidant/antioxidant status (malondialdehyde (MDA), total antioxidant capacity (TAC), superoxide dismutase (SOD), glutathione peroxidase (GPx), and catalase (CAT)) using commercially available kits (Northwest Life Sciences, Vancouver, WA, USA, and BioAssay Systems (EnzyChrom), San Francisco, CA, USA).

### 2.7. Histology of Ovary

The left ovary was carefully dissected and fixed in 10% formalin. The ovary was divided into small parts, processed, and stained with hematoxylin and eosin (H&E). Ovarian histological assessment was based on the number of cystic follicles (more than 12 cysts were considered as abnormal) and the presence of primary follicles, secondary follicles, and corpora lutea. The section was examined under an image analyzer (Olympus, Tokyo, Japan) at 40× magnification.

### 2.8. Statistical Analysis

All data obtained were analyzed using IBM SPSS version 22 (IBM Corp., Armonk, NY, USA). Distribution and variance of numerical data were evaluated by using whisker-box plot and Levene’s test, respectively. Data with normal distribution and homogenous variance were examined using one-way analysis of variance (ANOVA) followed by Tukey’s post-hoc test, and are presented as mean (standard error of mean, SEM). Categorical data was analyzed using Pearson’s chi-squared or Fisher’s exact tests, and are presented as percentages. A value of *p* < 0.05 was considered statistically significant.

## 3. Results

### 3.1. Phytochemical Screening and Liquid Chromatography–Mass Spectrometry (LC–MS) Analysis

**Figure 1:**
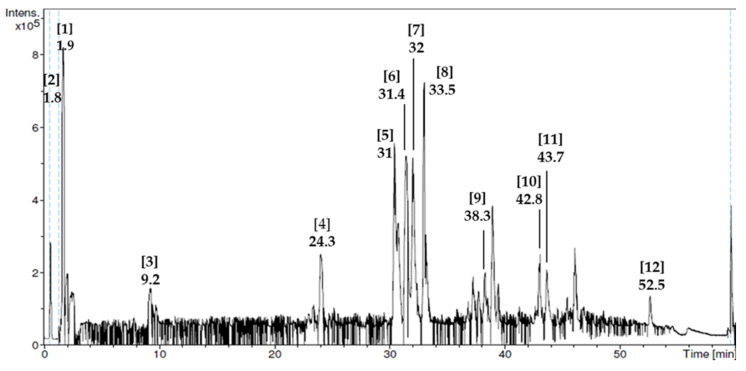
LC–MS chromatogram of royal jelly (peak assignments are listed in Table 2).

Phytochemical screening test revealed all nine phytochemical compounds found in RJ (Table 1) together with their reported bioactivities. From LC–MS analysis, we identified 12 non-volatile phytochemical compounds in RJ, and their reported biological activities are presented in Table 2. The compound with the highest mass spectrum was adenosine-5-monophosphate (348 *m/z*), while sebacic acid (185 *m/z*) had the lowest mass spectrum. The chromatogram and peak assignments of the compounds are shown in Figure 1.

### 3.2. Effect of Royal Jelly on Reproductive Hormones Levels in PCOS Rats

Testosterone levels were significantly higher in T and T+100RJ groups compared to the control group. However, the levels were significantly lower in T+200RJ and T+400RJ groups compared to the T group. Furthermore, E_2_ level was significantly higher in the T group compared to the control group. E_2_ levels in T+100RJ, T+200RJ, and T+400RJ groups were significantly lower compared to the T group, with the levels being almost similar to the control group. LH levels in T+200RJ and T+400RJ groups were significantly lower compared to control group. No significant differences of E_2_ and LH were observed among the T+100RJ, T+200RJ, and T+400RJ groups. Meanwhile, only the T+200RJ group showed a significantly lower level of LH compared to the T group. FSH levels in the T and T+100RJ groups were significantly lower compared to the control group. FSH level was significantly higher in the T+200RJ group compared to the T and T+100RJ groups (Table 3).

### 3.3. Effect of Royal Jelly on Regularity of Estrus Cycle in PCOS Rats

There was a significantly lower percentage of rats with regular estrus cycle in T and T+100RJ groups compared to the control group. However, the percentage of rats with regular estrus cycle were significantly higher in the T+200RJ group compared to the T and T+100RJ groups (Table 4).

### 3.4. Effect of Royal Jelly on Ovarian Oxidant/Antioxidant Status in PCOS Rats

Table 5 shows the ovarian oxidant/antioxidant status, which included MDA, TAC, SOD, GPx and CAT. The T group had a significantly higher MDA level with a lower level of TAC and GPx with respect to the control group. MDA levels in T+100RJ and T+200RJ groups were significantly lower compared to the T group and were insignificant when compared with the control group. T+400RJ had a significantly lower level of MDA compared to the control group. However, the TAC level was significantly lower in T+100RJ and T+400RJ groups compared to the control group, and a significantly higher TAC level was found in the T+200RJ group compared to the T and T+400RJ groups. SOD activity in the T+100RJ group was significantly higher compared to the control group, while in the T+200RJ group, SOD activity was significantly lower when compared to the T+100RJ group. In the T+200RJ group, GPx activity was significantly lower compared to the T group, being almost similar to the control group. However, there were no significant differences for CAT activity between all groups.

### 3.5. Effect of Royal Jelly on Ovarian Histology in PCOS Rats

**Figure 2:**
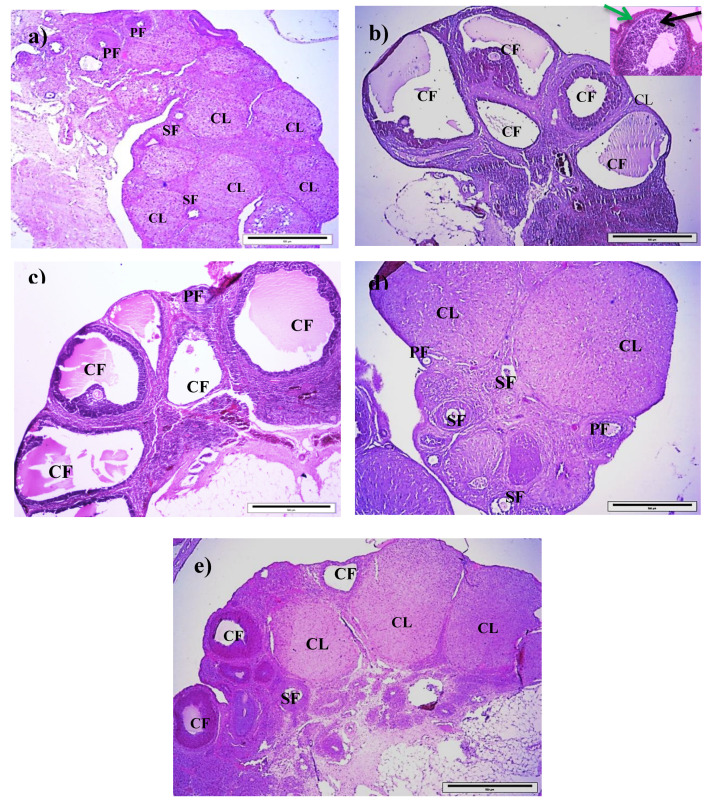
Representative photomicrograph of rat ovary showing normal follicular development with the presence of CLs, PFs, and SFs in control group (**a**), large CFs lining by thick theca cells (black arrow) and thin granulosa cells (green arrow) were predominant in T group (**b**), and T+100RJ group (**c**), while small CFs were observed in the T+400RJ group (**e**). However, in the T+200RJ group (**d**), these abnormal changes were improved. T: testosterone, RJ: royal jelly, CL: corpus luteum, CF: cystic follicle, PF: primary follicle, SF: secondary follicle. Photographs were taken using 40× (scale bar = 50 μm) objectives.

The ovary of control group exhibited normal follicular developments with the presence of corpora lutea, primary follicles, and secondary follicles (Figure 2a). After being induced with testosterone (T group), the ovary was embedded with large cystic follicle with thick theca cell lining and thin granulosa cells (Figure 2b). Figure 2c revealed the presence of cystic follicles after treatment with low dose of RJ (T+100RJ group). Histopathological observation in the T+200RJ group showed recovery of ovarian tissue with the presence of various stages of follicular development including corpora lutea, primary follicles, and secondary follicles, and the cysts mainly disappeared (Figure 2d). Meanwhile, in the T+400RJ group, the ovary had a reduced number of corpora lutea and reduced size of cystic follicles (Figure 2e).

From quantitative analysis (Table 6), the number of primary follicles was found to be significantly higher in the T, T+100RJ, and T+400RJ groups compared to the control group. In the T+200RJ group, the number was significantly lower compared to the T and T+100RJ groups but similar to the control group. However, the number of primary follicles was significantly higher in the T+400RJ group compared to the T+200RJ group. Numbers of secondary follicles in the T, T+100RJ, and T+400RJ groups were significantly lower compared to the control group. However, in the T+200RJ group, the number of secondary follicles was significantly higher compared to the T and T+100RJ groups; meanwhile, the number of secondary follicles was significantly lower in the T+400RJ group compared with the T+200RJ group. Number of corpora lutea was significantly lower in the T and T+400RJ groups compared to the control group. The number was significantly higher in the T+100RJ and T+200RJ groups compared with the T group. The T+200RJ group also had a significantly higher number of corpora lutea compared to the T+100RJ and T+400RJ groups. Numbers of cystic follicles were significantly higher in the T, T+100RJ, and T+400RJ groups compared to the control group. Meanwhile, the number of cystic follicles were significantly lower in the T+100RJ, T+200RJ, and T+400RJ groups compared with the T group. Number of cystic follicles in the T+200RJ group was also significantly lower compared to the T+100RJ group but similar to the control group.

## 4. Discussion

In the present study, we determined composition and anti-androgenic effect of RJ in a PCOS animal model. From our phytochemical screening test, we found that RJ used in the present study had flavonoids and phenols that were consistent with other studies performed on RJ from Jordan [47] and China [48]. Further analysis using LC–MS showed that it had compounds that have antioxidant properties such as caffeic acid derivatives [42] and anti-inflammatory properties such as sebacic acid [40]. To our knowledge, this is the first study to show the LC–MS profile of RJ.

In our animal experimental study, T propionate was injected into the rats to induce hyperandrogenism, which is one of the characteristics of PCOS. The significantly higher level of testosterone in the T group compared to the control group showed a successful induction of hyperandrogenism. This finding is consistent with previous studies that have reported on the high level of testosterone in rats after being induced by T propionate [49], letrozole [50], and dehydroepiandrosterone [51]. Another animal study in prenatally androgenized adult female offspring showed significantly increased testosterone levels when compared to control rats [52]. In a clinical study, PCOS patients exhibited a higher level of total testosterone as compared to normal women [53]. On the other hand, T level was significantly lower in T+200RJ and T+400RJ groups compared to the T group, which is consistent with another study [12]. This might suggest that RJ at 200 and 400 mg/kg is able to lower T level in this PCOS animal model.

The present study also showed a significantly higher E_2_ level in the T group compared to the control group, which is similar with other studies. For example, significant increase of E_2_ level was also found in PCOS mouse ovary after treatment with dehydroepiandrosterone [54] and in PCOS patients [55] compared to control group, which might contribute to unfavorable conditions for the development of follicles. It is possible that high E_2_ level was attributed to the concomitant high level of testosterone, as it can be converted to form E_2_. Interestingly, E_2_ level was significantly lower in all groups treated with RJ compared to the T group, which is consistent with other studies using other natural products such as bee venom in E_2_-valerate-induced rats [56] and *Commiphora wightii* in dehydroepiandrosterone-induced PCOS rats [51]. The low E_2_ levels found in the present study might have been caused by concomitant low levels of testosterone. Furthermore, the non-significant differences in E_2_ levels among T+100RJ, T+200RJ, and T+400RJ groups might suggest that the effect of RJ is not dose-dependent.

LH level in the T group was not significantly different from the control group, suggesting that this PCOS animal model did not have an effect on LH level. However, LH level is significantly higher in PCOS women than normal women [57] and it occurs in about 60% of women with PCOS [58]. Elevated LH level results in high production of androgen by theca cells in ovaries [59]. Therefore, the normal level of LH in T group might suggest that the high T level is not attributable to LH level but may be due to high synthesis of T, which needs further study. However, LH level was significantly lower in the T+200RJ group compared to the control group, which is in agreement with a previous study in which administration of myoinositol, an insulin sensitizer, in PCOS patients reduced LH level and LH/FSH ratio as well as improved menstrual cycle [60]. In contrast, in another study, LH level was significantly increased in infertile men treated with RJ [61]. Furthermore, in a study of male rats, supplementation of ofloxacin concomitant with RJ led to elevated level of LH [62]. Hence, the low LH level in rats treated with RJ in this animal model of the present study may suggest a low secretion of LH by anterior pituitary which needs further study. FSH level in T group that was significantly lower compared to control group, which is similar with other studies in which serum FSH level was low in androgen-sterilized female rats compared with normal rats [63]. The low FSH level in the T group might have been due to its concomitant high E_2_ level that could lead to low FSH secretion by the anterior pituitary. However, FSH level was significantly higher in the T+200RJ group compared to T group which could be attributed to its concomitant low E_2_ level.

The T group had a significantly lower percentage of regular estrus cycle compared to the control group, suggesting successful induction of hyperandrogenism that induced an irregular estrus cycle. This is in accordance with other studies that administered testosterone in prenatal period [52] and in immature female rats, resulting in irregular estrus cycle [25]. The irregular estrus cycle is suggested due to the significantly high T, high E_2_, and low FSH levels found in the T group. This is supported by another study where the irregular estrus cycle in androgenized transgenic mice exhibited an alteration in hypothalamic–pituitary–gonadal axis function [64]. However, in the T+200RJ group, its percentage of regular estrus cycle was significantly higher than T group, which is in agreement with other previous studies [17,65]. The improved estrus cycle in the T+200RJ group could be explained by the improvement in its T, E_2_, LH, and FSH levels. Furthermore, this finding might indicate that administration of RJ has the capability to modulate estrogenic activity that could ameliorate the impaired reproductive function in PCOS. In addition, the estrogenic property might also be attributed by the presence of 10-HDA and HDAA in RJ, which have been reported to have estrogenic activity [66].

In the present study, the ovarian MDA level, a marker of lipid peroxidation, was significantly higher in the T group compared to the control group, which is in agreement with a clinical study in which MDA level was significantly higher in PCOS patients compared to control patients, suggesting the presence of OS in PCOS [67]. Hyperandrogenemia is suggested to be the reason for higher MDA level or imbalance in oxidant/antioxidant status, even though the exact mechanism is not clearly understood. The significantly lower ovarian MDA levels in the T+100RJ and T+200RJ groups might suggest the ability of RJ to ameliorate OS in rat ovary, which corresponds to other studies [68,69].

Ovarian TAC level was significantly lower in the T group compared with the control group, which is consistent with another study that reported decreased serum TAC level in women with PCOS [67] and low serum TAC level in E_2_-vealerate-induced PCOS rats [70]. This low level of TAC might explain the high ovarian MDA level in the T group. However, ovarian TAC level in the T+200RJ group was significantly higher compared to the T group, which might be attributable to the lower ovarian MDA level found in the T+200RJ group. This finding is similar to other studies, whereby RJ augmented the TAC level on paclitaxel-induced cardiotoxicity in rats [71] and in patients with insulin resistance type 2 diabetes mellitus [72]. In the present study, activity of ovarian GPx in the T group was significantly higher compared to the control group, which could be a result of up-regulation or increased synthesis of GPx as a compensatory mechanism to overcome OS. This finding is consistent with a previous study that reported increased GPx activity in PCOS patients [53]. However, the activity of GPx was significantly decreased in the T+200RJ group, which might suggest the ability of RJ at a dose of 200 mg/kg to reduce the increased activity of GPx in this PCOS animal model. For ovarian CAT activity, no significant difference was found between T and control groups, which is similar to other study whereby there was no difference for CAT activity in PCOS patients as compared to controls [73]. There were no changes of CAT activities in T+100RJ, T+200RJ, and T+400RJ groups, which might suggest that RJ has no effect on CAT activity. However, the increased SOD activity in the T+100RJ group might suggest that RJ at a dose of 100 mg/kg possibly could up-regulate or increase the synthesis of SOD, which requires further study. The significant changes observed on oxidant/antioxidant markers in rat from the T+200RJ group might indicate that RJ at the dose of 200 mg/kg exhibits an optimal antioxidant property that counteracts OS in this PCOS animal model. The antioxidant property of RJ has also been reported in a study, as RJ has a protective oxidative effect against cisplatin-induced testicular damage in rats [12], as well as in yeast *Saccharomyces cerevisiae* cells, in which it could reduce intracellular oxidation [74]. The improved oxidant/antioxidant status could also be attributed to some phytochemical compounds found in RJ from the LC–MS analysis that have antioxidant properties such as caffeic acid [42] and dimethoxycinnamic acid [75] derivatives.

Histologically, the T group had a significantly higher number of primary and cystic follicles, as well as a significantly lower number of secondary follicles and corpora lutea compared to the control group, suggesting the establishment of PCOS characteristics in this animal model. A higher number of primary follicles may suggest the presence of a high number of retarded primary follicles that did not develop into secondary follicles and corpora lutea, as supported by the concomitant low numbers of secondary follicles and corpora lutea in the T group. It is suggested that elevated androgen concentration stimulates follicular growth by increasing FSH receptor expression, leading to formation of multiple follicles. However, at a low FSH level, the growth of follicles is restricted, which in turn leads to formation of atretic and cystic follicles [76]. It has been suggested that androgen also modifies the response of follicles in the ovary to gonadotropins, giving rise to the changes found in polycystic ovaries [76].

Interestingly, all these ovarian histological changes were significantly improved in the T+200RJ group compared to the T group, although there were improvements for a number of corpora lutea in the T+100RJ group and a number of cystic follicles in the T+100RJ and T+400RJ groups, suggesting that RJ at 200 mg/kg/day is an optimum dose for the improvement of ovarian histology in this PCOS animal model. Furthermore, this histological finding may also explain the improved estrus cycle and reproductive hormonal levels found in the T+200RJ group. Studies have also speculated that the increase in OS has led to anovulation in terms of reduced granulosa cell luteinization and oocyte maturation [77]. Therefore, it is plausible to suggest that this antioxidant effect of RJ possibly could explain the improved ovarian histology and function, as well as the estrus cycle in the T+200RJ group. We would also like to speculate that the beneficial ovarian changes found in the present study might have been due to the action of royalactin, a 57 kDa protein that is present in RJ, which can stimulate normal ovarian development in queen bees [78]. In addition, the improvement in the ovary could also be attributed to its phytochemical compounds such as 10-HDA and HDAA found in RJ, which can modulate estrogenic activity [66].

## 5. Conclusions

RJ at the dose of 200 mg/kg for 4 weeks significantly improved reproductive hormone levels (T, E_2,_ FSH, LH), estrus cycle regularity, ovarian oxidant-antioxidant status (MDA, TAC, GPx), and ovarian histology in a PCOS animal model. These effects could be attributed partly to the combined anti-androgenic effect of RJ, which possess phytochemical and bioactive antioxidant compounds, and requires further study to determine its exact mechanism of action.

## Figures and Tables

**Table 1:** Phytochemical screening of royal jelly and bioactivities of the compounds.

Compounds	Intensity	Activities
Alkaloids	+	Anti-inflammatory effect, anti-asthmatic and anti-anaphylactic activities [27].
Phenols	+	Antibacterial and anti-hemolytic activities [28].
Flavonoids	++	Antimicrobial, anti-proliferative [29], and antioxidant activities [27].
Glycosides	++	Preservative [29].
Resins	++	Antimicrobial activity [30].
Saponins	++	Anti-hypercholesterolemia and antibiotic activities [28].
Tannins	++	Anti-hemolytic activity [28].
Terpenoids	++	Cytotoxic, anti-fungal, antibacterial, and anti-inflammatory activities [29].
Xanthoproteins	+	No activity reported.

Positive sign (+) indicates the presence of compound while double positive sign (++) indicates the presence of compound with higher intensity of color changes. Each test was carried out in triplicate (*n* = 3 per test).

**Table 2:** LC–MS analysis of royal jelly and bioactivities of the compounds.

No.	Compound Name	Retention Time (min)	Mass Spectrum (*m/z*)	Bioactivities
1	Adenosine-5-monophosphate	1.9	348	Induces neurite outgrowth (neuritegenesis) [31], modulates gonadotrophin-releasing hormone (GnRH) release and estrus activity [32].
2	Guanosine-5-monophosphate	1.8	195	Regulates oocytes maturation [33].
3	Adenosine	9.2	220	Anticonvulsant, anti-ischemic, analgesic, and neuroprotective activities [34].
4	d-pantothenic acid hemicalcium salt	24.3	227	Improves sperm motility and maturity [35], reduces cardiovascular risk [36] and inflammation [37]
5	Homovanillic acid	31	155	No activity reported.
6	Carboxylic acid	31.4	151	Required for formation of other important components in body such as fatty acid [38].
7	Sebacic acid	32	185	Anti-diabetic [39] and anti-inflammatory effects [40].
8	Methoxybenzoic acid derivative	33.5	187	No activity reported.
9	Baclofen	38.3	237	Enhances GnRH mRNA level [41]
10	Caffeic acid derivative	42.8	206	Antioxidant [42] and anti-implantation activities during early pregnancy in mice [43].
11	Dimethoxycinnamic acid derivative	43.7	295	Inhibits prion propagation [44] and alpha-synuclein amyloid aggregation [45]
12	Phosphocholine derivative	52.5	206	One of the C-reactive protein-binding targets to carry out immunologic response [46]

LC–MS: Liquid chromatography–mass spectrometry, RJ: royal jelly, RT: retention time.

**Table 3:** Effect of royal jelly on reproductive hormones in polycystic ovarian syndrome (PCOS) rats.

Groups	T (ng/mL)	E_2_ (pg/mL)	LH (ng/mL)	FSH (ng/mL)
Control	1.99 (0.07)	52.73 (1.3)	3.72 (0.05)	81.23 (1.8)
T	3.35 (0.18) ^a^	88.89 (2.98) ^a^	3.69 (0.03)	56.52 (3.09) ^a^
T+100RJ	2.95 (0.25) ^a^	60.15 (1.39) ^b^	3.56 (0.03)	62.95 (3.15) ^a^
T+200RJ	2.07 (0.06) ^b,c^	57.43 (3.74) ^b^	3.45 (0.19) ^a,b^	85.39 (1.7) ^b,c^
T+400RJ	2.96 (0.08) ^b,d^	60.96 (1.8) ^b^	3.49 (0.2) ^a^	70.49 (8.56)

Data are presented as mean (SEM), (*n* = 8 rats/group). T: testosterone, E_2:_ estradiol, LH: luteinizing hormone: FSH: follicle-stimulating hormone, RJ: royal jelly. ^a^ *p* < 0.05 compared with control group, ^b^ *p* < 0.05 compared with T group, ^c^ *p* < 0.05 compared with T+100RJ group, ^d^ *p* < 0.05 compared with T+200RJ group (one-way ANOVA followed by Tukey’s post hoc test).

**Table 4:** Rats with regular estrus cycle in all experimental groups.

Groups	Rats with Regular Estrus Cycle (%)
Control	87.5
T group	25.0 ^a^
T+100RJ	25.0 ^a^
T+200RJ	87.5 ^b,c^
T+400RJ	50.0

Data are presented in percentage (%; *n* = 8 rats/group). T: testosterone, RJ: royal jelly. ^a^ *p* < 0.05 compared with control group, ^b^ *p* < 0.05 compared with T group, ^c^ *p* < 0.05 compared with T+100RJ group (Fisher’s exact test).

**Table 5:** Ovarian oxidant/antioxidant status in all experimental groups.

Group	MDA (nmol/mg Protein)	TAC µM Trolox Equivalents	SOD (U/mg Protein)	GPx (U/mg Protein)	CAT (U/mg Protein)
Control	0.09 (0.18)	0.25 (0.01)	1.92 (0.32)	34.1 (4.75)	63.61 (0.63)
T	0.39 (0.14) ^a^	0.10 (0.01) ^a^	2.93 (0.34)	100.69 (14.39) ^a^	64.47 (0.41)
T+100RJ	0.15 (0.05) ^b^	0.12 (0.01) ^a^	3.67 (0.15) ^a^	66.76 (21.81)	59.07 (4.16)
T+ 200RJ	0.15 (0.07) ^b^	0.21 (0.03) ^b^	2.19 (0.15) ^c^	40.09 (5.89) ^b^	54.10 (4.46)
T+400RJ	0.3 (0.06) ^a^	0.12 (0.03) ^a,d^	2.57 (0.53)	54.15 (14.53)	53.10 (7.83)

Data are presented as mean (SEM; *n* = 8 rats/group). T: testosterone, RJ: royal jelly, MDA: malondealdehyde, TAC: total antioxidant capacity, SOD: superoxide dismutase, CAT: catalase, GPx: glutathione peroxidase. ^a^ *p* < 0.05 compared with control group, ^b^ *p* < 0.05 compared with T group, ^c^ *p* < 0.05 compared with T+100RJ group, ^d^ *p* < 0.05 compared with T+200RJ group (one-way ANOVA followed by Tukey’s post hoc test).

**Table 6:** The number of primary and secondary follicles, number of corpora lutea, and number of cystic follicles in all experimental groups.

Variables	No. of Primary Follicles	No. of Secondary Follicles	No. of Corpora Lutea	No. of Cystic Follicles
Control	3.25 (0.37)	7.88 (0.8)	6.64 (0.93)	1.13 (0.3)
T	7.25 (0.53) ^a^	2.25 (0.36) ^a^	3.13 (0.35) ^a^	9.25 (0.77) ^a^
T+100RJ	5.88 (0.40) ^a^	2.38 (0.42) ^a^	4.75 (0.59) ^b^	5.50 (0.42) ^a,b^
T+200RJ	3.25 (0.37) ^b,c^	7.00 (0.57) ^b,c^	8.75 (0.36) ^b,c^	3.00 (0.423) ^b,c^
T+400RJ	6.00 (0.33) ^a,d^	2.13 (0.3) ^a,d^	3.75 (0.45) ^a,d^	4.38 (0.26) ^a,b^

Data are presented as mean (SEM; *n* = 8 rats/group). T: testosterone, RJ: royal jelly. ^a^ *p* < 0.05 compared with control group, ^b^ *p* < 0.05 compared with T group, ^c^ *p* < 0.05 compared with T+100RJ group, ^d^ *p* < 0.05 compared with T+200RJ group (one-way ANOVA followed by Tukey’s post hoc test).
